# PReS-FINAL-2320: Different clinical presentation of takayasu arthritis: case report of two pediatric patients

**DOI:** 10.1186/1546-0096-11-S2-P310

**Published:** 2013-12-05

**Authors:** I Rukavina, I Malčić, M Frković, M Jelušić

**Affiliations:** 1 Clinical Hospital Center Zagreb, Zagreb, Croatia

## Introduction

Takayasu arteritis (TA) is a granulomatous large vessel vasculitis that involves the aorta, its major branches and pulmonary arteries. Diagnosis of TA during childhood remains challenging due to the non-specific symptoms. It primarily affects East Asian women in their second or third decade of life but is well known to affect all ethnicities across the world. Given its systemic nature, Takayasu's arteritis has multiorgan involvement, with the majority of disease morbidity related to the cardiovascular, central nervous, and renal systems. Despite the increasing identification of children and adolescents with TA, reports of disease in childrens population are still scarce.

## Objectives

Objective of our research was analysing of clinical, laboratorial and radiological characteristics, organ involvment, type of therapies and outcome of patients diagnosed with TA.

## Methods

we reviewed the medical records of all patients aged 1-18 years who were diagnosed with TA (according to EULAR/PRINTO/ PRES criteria) during the period 2002 - 2012, at the Department of Paediatrics, University Hospital Centre Zagreb, University of Zagreb School of Medicine.

## Results

TA was diagnosed at two patients, both girls, aged 14 and 16 years. First patient presented with fatigue, chest and left arm pain, especially during physical activity. On admission, she had absent the left brachial and radial pulses, bruits and increased erythrocyte sedimentation rate (ESR), C-reactive protein (CRP) level and fibrinogen. Magnetic resonance angiography (MRA) showed narrowing in ascending aorta, brachiocephalic, right common carotid, left subclavian, left vertebral arteries and descending aorta. Heart chateterisation with coronarography showed high-grade stenosis of left coronary arthery. She was treated with puls corticosteroid therapy (3 consecutive days) followed by corticosteroids orally and pulse cyclophosphamide therapy (6x) after what clinical picture has improved and laboratory findings has become normalized.

Second patient presented with hypertension. She did not have any symptoms of disease and tolerate efforts normally. On admission she had absent lower extremities pulses. MSCT aortography showed narrowing of abdominal aorta 10 centimetres long with poststenotic dilatation and aneurysm. Narrowing of upper right renal arteria was seen as well. Positron emission tomography - computed tomography (PET-CT) revealed areas of inflammation in the abdominal aorta and markers of inflammation in blood were also elevated (ESR, CRP, fibrinogen). Patient was treated with the aorto-aortic grafting, metotrexate and corticosteroids orally. At the last follow up antihypertensive therapy has been modified because girl is still having a hypertension.

## Conclusion

TA is rare in children; however, childhood TA must be considered in children who present with non-specific systemic symptoms, chest disease, hypertension and increased acute phase reactants.

## Disclosure of interest

None declared.

